# Genomic pathways modulated by Twist in breast cancer

**DOI:** 10.1186/s12885-016-3033-3

**Published:** 2017-01-13

**Authors:** Farhad Vesuna, Yehudit Bergman, Venu Raman

**Affiliations:** 1 Division of Cancer Imaging Research, Department of Radiology, Johns Hopkins University School of Medicine, Baltimore, MD 21205 USA; 2 Department of Oncology, Johns Hopkins University School of Medicine, Baltimore, MD 21287 USA; 3 Department of Pathology, University Medical Center Utrecht, Utrecht, The Netherlands

**Keywords:** Breast cancer, Genomics, Twist, Orthotopic, Xenograft, Mouse model, Pathways, Genes, Microarray, Human

## Abstract

**Background:**

The basic helix-loop-helix transcription factor TWIST1 (Twist) is involved in embryonic cell lineage determination and mesodermal differentiation. There is evidence to indicate that Twist expression plays a role in breast tumor formation and metastasis, but the role of Twist in dysregulating pathways that drive the metastatic cascade is unclear. Moreover, many of the genes and pathways dysregulated by Twist in cell lines and mouse models have not been validated against data obtained from larger, independant datasets of breast cancer patients.

**Methods:**

We over-expressed the human Twist gene in non-metastatic MCF-7 breast cancer cells to generate the estrogen-independent metastatic breast cancer cell line MCF-7/Twist. These cells were inoculated in the mammary fat pad of female severe compromised immunodeficient mice, which subsequently formed xenograft tumors that metastasized to the lungs. Microarray data was collected from both in vitro (MCF-7 and MCF-7/Twist cell lines) and in vivo (primary tumors and lung metastases) models of Twist expression. Our data was compared to several gene datasets of various subtypes, classes, and grades of human breast cancers.

**Results:**

Our data establishes a Twist over-expressing mouse model of breast cancer, which metastasizes to the lung and replicates some of the ontogeny of human breast cancer progression. Gene profiling data, following Twist expression, exhibited novel metastasis driver genes as well as cellular maintenance genes that were synonymous with the metastatic process. We demonstrated that the genes and pathways altered in the transgenic cell line and metastatic animal models parallel many of the dysregulated gene pathways observed in human breast cancers.

**Conclusions:**

Analogous gene expression patterns were observed in both in vitro and in vivo Twist preclinical models of breast cancer metastasis and breast cancer patient datasets supporting the functional role of Twist in promoting breast cancer metastasis. The data suggests that genetic dysregulation of Twist at the cellular level drives alterations in gene pathways in the Twist metastatic mouse model which are comparable to changes seen in human breast cancer﻿s. Lastly, we have identified novel genes and pathways that could be further investigated as targets for drugs to treat metastatic breast cancer.

**Electronic supplementary material:**

The online version of this article (doi:10.1186/s12885-016-3033-3) contains supplementary material, which is available to authorized users.

## Background

Despite significant advancements in understanding the biology of breast cancer progression, there are still many aspects of this disease that are not well understood, such as the metastatic process, which is the largest cause of mortality in breast cancer patients. There has been a concerted effort to understand the mechanics of this process in order to provide new opportunities to design targeted interventions to inhibit or prevent metastatic progression. A number of in vitro and in vivo mouse preclinical models have been used to identify key components that are drivers of the metastatic process. However, given the heterogeneity of breast cancer and the relevance of the model used to the human disease, there are significant quantitative and qualitative differences between human breast tumors and preclinical mouse models. One possible alternative is to define gene-specific alterations and higher order pathways that are dysregulated both in human breast cancers and in preclinical mouse models and cell lines. If the dysregulated gene signatures identified from breast cancer patient samples can be functionally validated in cell lines and in xenograft models, this may help in understanding breast cancer progression. Simultaneously, if genetic changes that contribute to breast cancer progression can be catalogued using cell line and xenograft-based models, it may be possible to derive gene signatures that, at least partly, replicate the progression of breast cancer in humans. Importantly, this could be accomplished in cell lines that ordinarily do not express these genes. It may also be possible to altogether avoid mouse models in certain types of breast cancers if cell lines are demonstrated to be validated surrogates of the cancer.

In our quest to genetically define breast cancer progression, we identified the gene Twist (TWIST1), which promotes the metastatic process in humans. Twist is a transcription factor belonging to the basic helix-loop-helix (bHLH) family of proteins and is essential for normal vertebrate development [1–3]. Twist is overexpressed in breast [4, 5], gastric [6], and prostate cancers [7], as well as in melanomas [8], gliomas [9], osteosarcomas [10], and rhabdomyosarcomas [11]. Functionally, Twist overexpression promotes breast cancer by increasing angiogenesis and chromosomal instability [4, 12], and by downregulating E-cadherin [13] and estrogen receptor [14]. We have also demonstrated that Twist promotes the breast cancer stem cell phenotype by regulating CD24 [15]. Even though the functional roles of Twist in cancer biogenesis are well documented, there is, however, little evidence presented in the literature characterizing the global dysregulation of genes and pathways brought about as a direct consequence of Twist overexpression [4, 5]. Here, we show by establishing a new mouse orthotopic xenograft model, that breast tumors overexpressing Twist are highly metastatic to the lungs. We also demonstrate that the genes and pathways altered in our Twist over expressing cell line and metastatic xenograft mouse model recapitulate some of the observations seen in human breast tumors. Our data establishes a new mouse model of metastatic breast cancer that can be used to test the efficacy of therapies for breast cancer treatment.

## Methods

### Cell lines and animal experiments

MCF-7 cell line was originally obtained from ATCC (Manassas, VA). MCF-7/Twist cell line was created earlier [4] and constitutively over-expresses Twist. One million MCF-7/Twist cells were injected into the second left mammary fat pad of female SCID mice which were 4–6 weeks old. Breast tumors were allowed to grow for a period of 2 months after which the tumors were resected in a sterile environment. MCF-7/Twist primary tumor explants were then grown in tissue culture till cell lines were obtained. Fibroblast contamination was removed by using low concentrations of G418 for short periods. After a further period of 2 months, mice were sacrificed and lungs were examined for metastases. Lungs were minced under sterile conditions and made into a single cell suspension by dispersing the tissue using collagenase and DNase I treatment. Single cells were then plated in cell culture dishes and expanded till enough MCF-7/Twist metastatic cells were obtained for RNA extraction. RNA was extracted from all cell lines in the early part of their growth for performing microarray analysis.

### Microarray analysis

The RNA samples were converted into double stranded cDNA which was subsequently transcribed into biotinylated complementary RNA. After purification and fragmentation, the cRNA was hybridized to GeneChip Human Genome U133 Plus 2.0 array chips (Affymetrix, Santa Clara, CA), and scanned using an Affymetrix GeneChip Scanner 3000 with default parameters to obtain gene expression profiles. Affymetrix CEL file data was extracted and their data was normalized using RMA (Robust Multi-array Average), and averaged over biological replicates, creating the quantile-normalized log2 transcript signal values used in subsequent ANOVA analyses. Analysis was performed using Genomic Suite Software 6.6 (Partek, St. Louis, Missouri).

Genes whose expression differed by at least 1.5 fold from the median in at least 20% of the arrays were retained. We identified genes that were differentially expressed among the classes using a multivariate permutation test [16]. We used the multivariate permutation test to provide 90% confidence that the false discovery rate was less than 10%. The false discovery rate is the proportion of the list of genes claimed to be differentially expressed that are false positives. The multivariate permutation test is non-parametric and does not require the assumption of Gaussian distributions.

### Hierarchical clustering analysis

To confirm the relationship between the various classes, we performed unsupervised hierarchical clustering of classes using the UPGMA (Unweighted Pair Group Method with Arithmetic Mean) method.

### Volcano plots

To further study the gene expression differences among the different samples, we visualized the data by volcano plots. The log_2_ fold-change in expression is seen on the x-axis with red depicting mRNAs that are up-regulated and green depicting mRNAs that are down-regulated. The y-axis depicts the -log_10_ significance between the classes. The horizontal line depicts *P* = 0.05 and values above were considered statistically significant.

## Results

### Experimental study design and bioinformatics analysis

**Fig. 1:**
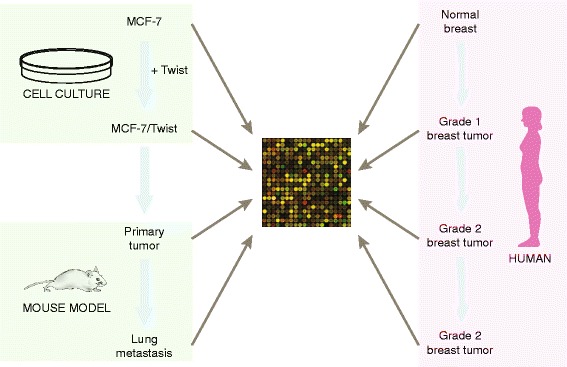
Overall methodology of the approach adopted in this research work. Twist was stably overexpressed in MCF-7 cells creating the MCF-7/Twist cell line. These Twist overexpressing cells were orthotopically inoculated in female SCID mice to form breast tumors which subsequently gave rise to lung metastases. Microarray analysis was performed at all stages of this process - the parental MCF-7 cell line, MCF-7/Twist cell line, primary breast xenograft tumors, and lung metastasis. In parallel, human breast tumors of grades 1–3 were also analyzed for gene expression

To study genomic pathways that are disrupted by Twist expression in breast cancer, we designed an experimental plan that encompasses cell culture, a mouse xenograft model, and human breast cancer samples (Fig. 1). We stably overexpressed Twist in MCF-7 breast cancer cells to establish the MCF-7/Twist cell line. Functional analysis revealed that this cell line is aggressive, forms large primary tumors, and is metastatic when implanted orthotopically in the breast of SCID mice [4]. Inoculation of MCF-7/Twist cells into the mammary fat pad of SCID mice gave rise to tumors in all mice (*n* = 8) within 1–2 months. These tumors were surgically resected two months after inoculation and expanded into cell lines. After an additional period of 1–2 months, all mice had developed lungs metastases, which were confirmed by histological analysis of the lungs. We thus established an orthotopic mouse xenograft model of breast cancer that mirrors the ontogeny of tumor formation and progression of lung metastasis in humans (Fig. 1). In order to analyze the genes and pathways that were dysregulated by Twist in our models, we studied RNA transcripts from the parental MCF-7 and the Twist over expressing MCF-7/Twist cell lines, primary orthotopic tumors and lung metastases in mice. RNA was analyzed by microarray analysis.

A total of 54,675 probe sets were obtained from microarray analysis after normalization. Of these, using the NetAffyx Annotation Release 35 dataset, probe sets of unknown origin (8,841), controls (62), and probes mapping to multiple gene assignments (2,796) were discarded. The remaining 42,976 probe sets were mapped to 21,655 genes.

**Fig. 2:**
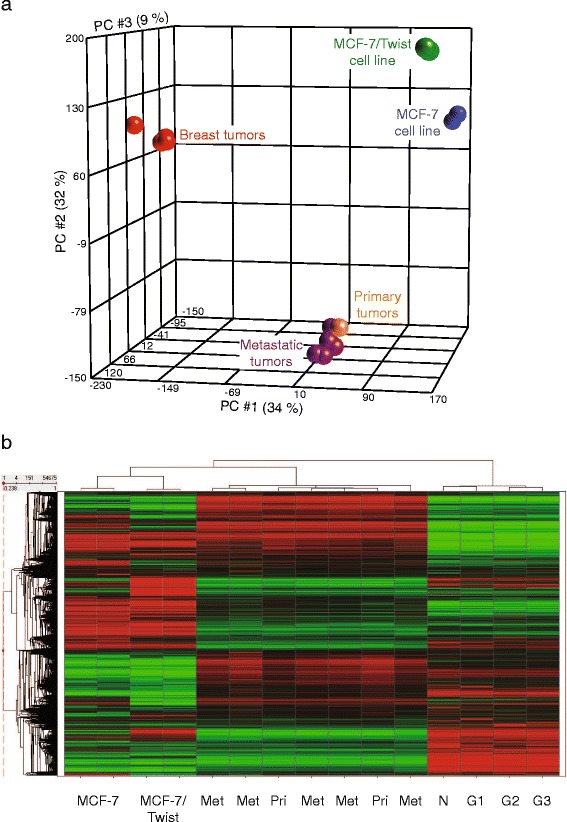
**a** PCA (principal component analysis) plot displaying relative variation between all samples used in the study. Principal components 1, 2, and 3 account for 34%, 32%, 9% of the variability, respectively. **b** Heat map comparing the gene expression levels of MCF-7, and MCF-7/Twist cell lines, primary breast tumors (Pri), lung metastases (Met), and human breast tumors (grade 1 = G1, grade 2 = G2, grade 3 = G3). Normal breast gene expression levels are indicated by N

To study the overall variation between the sample classes, we initially performed unsupervised PCA analysis (Fig. 2a). PCA analysis showed that the parental MCF-7 clustered with the MCF-7/Twist cell line, while the xenograft tumors clustered with the metastatic tumors, and the samples from breast cancers formed an independent tightly knit cluster. The cell lines MCF-7 and MCF-7/Twist clustered relatively further apart from one another indicating that over expression of Twist caused large genomic changes in the parental MCF-7 cells. From the close clustering of the orthotopic primary and metastatic tumors, we surmise that the gene alterations induced by Twist expression were attenuated to some degree in the in vivo mouse model.

Hierarchical clustering analysis was performed on the MCF-7 and MCF-7/Twist cell lines, on the mouse primary xenograft tumors, and lung metastases and on the human breast tumors (Fig. 2b). From the hierarchical clustering analysis, we determined the closest relationship observed was within human tumor samples and the mouse xenograft samples (Fig. 2b). We also observed the clustering of cell lines according to their propensity to metastasize with MCF-7 cell being farthest from MCF-7/Twist lung metastases.

### Gene expression analysis

**Fig. 3:**
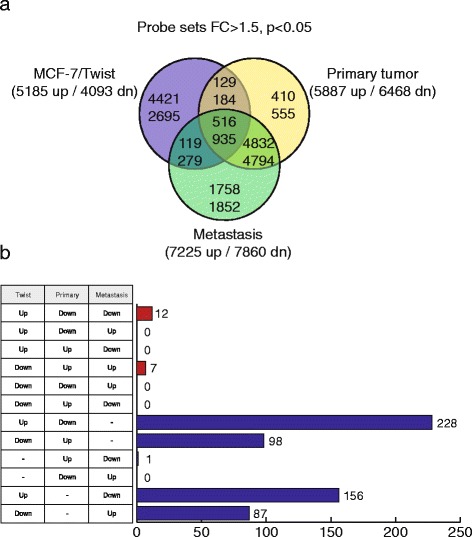
**a** Venn diagram displaying the probe sets and genes up and down-regulated in the various classes. Only genes that varied significantly (fold-change >1.5, and P < 0.05) are displayed. Numbers on top row indicate probe sets with increased expression, while numbers in lower row indicate probe sets with decreased expression. **b** Combined table and histogram displaying the number of genes that were expressed differentially in the three classes. The table indicates the direction of expression and the histogram indicates the number of genes expressed in the classes

We compared mRNA expression between all three groups (MCF-7/Twist cells, primary tumors, and lung metastases) using parental MCF-7 cells as controls (Fig. 3a). There were a total of 1,451 probe sets that were common between all three datasets (MCF-7/Twist cells, MCF-7/Twist primary tumors and metastases) of which 516 were up-regulated and 935 were down-regulated (Fig. 3a). See Additional File 1 for the complete list of genes up and down-regulated in all three classes. We validated the top two genes by quantitative reverse transcription polymerase chain reaction (qRT-PCR) in these two datasets and the results are shown in Additional File 2.

Next, we analyzed the expression data for genes that were differentially expressed in the three groups (Fig. 3b). When comparing multidirectional gene expression in three classes, 12 probe sets were up-regulated in MCF-7/Twist while being down-regulated in the primary tumors and metastases. On the other hand, we found 7 probe sets down-regulated in MCF-7/Twist that were up-regulated in primary tumors and metastases. Subsequently, we compared multidirectional gene expression across two classes. 228 probe sets were up-regulated in the MCF-7/Twist cell line while being down-regulated in primary tumors. Similarly, 156 probe sets were up-regulated in Twist cells while down-regulated in metastatic tumors. Conversely, 98 probe sets were down-regulated in MCF-7/Twist cells while they were up regulated in primary tumors. Similarly, 87 probe sets were down-regulated in Twist cells while being up-regulated in metastatic tumors. Overall, we found that Twist caused larger changes in gene expression in the MCF-7/Twist cell line as compared to changes caused in the primary or metastatic tumor classes. Complete probe sets are listed in Additional File 3.

A total of 9,278 probe sets (6,478 genes) had their expression significantly altered (FC > 1.5, P < 0.05) in the MCF-7/Twist cell line (5,185 probe sets/3,497 genes up-regulated and 4,093 probe sets/2,981 genes down-regulated). The probe sets differentially expressed (up and down-regulated) in MCF-7/Twist cells are listed in Additional file 4. The top two genes in both directions were validated by qRT-PCR and the result is shown in Additional file 5.

In the primary tumors, 12,355 probe sets (10,182 genes) had altered expression compared to MCF-7 (5,887 probe sets/4,771 genes up-regulated and 6,648 probe sets/5,411 genes down-regulated) (See Additional File 6 for complete list). The qRT-PCR validation is displayed in Additional File 7.

In comparison to MCF-7 cells, the metastatic tumors had 15,085 probe sets (12,183 genes) that were significantly altered of which 7,225 probe sets/5,825 genes were up-regulated and 7,860 probe sets/6,358 genes were down-regulated (Additional File 8). The top genes from the up and down-regulated datasets were validated by qRT-PCR (Additional File 9).

As could be expected, bioinformatics analysis indicated that the largest overlap of 11,077 probe sets occurred between the primary and metastatic tumors (5,348 up- and 5,729 down-regulated). This is displayed in Fig. 3a, and the complete list is in Additional File 10. Additional File 11 displays the results of the validation of this data by qRT-PCR.

There were 1,849 probe sets in common between the MCF-7/Twist cell line and metastasis (635 up- and 1,214 down-regulated), the complete list of which is displayed in Additional File 12. Validation of the top 2 genes (up and down-regulated) was performed by qRT-PCR and is shown in Additional File 13.

Figure 3a also exhibits that the least overlapping gene signature was between the MCF-7/Twist cells and primary tumor classes, where 1,764 probe sets were regulated commonly (645 up- and 1,119 down-regulated). All the expressed probe sets are displayed in Additional File 14 and the validation performed by qRT-PCR is shown in Additional File 15.

Intriguingly, the MCF-7/Twist cell line had more down-regulated probe sets in common with the metastatic tumors and more up-regulated probe sets in common with the primary tumors.

Highly expressed transcripts in MCF-7/Twist cells include EDIL3, which has, so far, not been found to be involved in cancer development but is involved in immune system-dependent engulfment of apoptotic cells [17]. Another high expressing transcript includes LDHB, which is involved in triple negative breast cancer [18]. ESR1 (estrogen receptor alpha), GATA3 (GATA Binding Protein 3), and CDH1 (E-cadherin) are amongst the genes most down-regulated by Twist expression and shown to be involved in the progression of breast cancer earlier [13, 14, 19]. Genes that were up-regulated in primary and metastatic tumors included SETD5 (SET domain containing 5) and TFAP2A (transcription factor AP-2 alpha). Intriguingly, very little is known about SETD5 in breast cancer progression and metastasis [20], while TFAP2A is involved in both sporadic and hereditary breast cancer [21, 22]. SLC6A14 (solute carrier family 6, member 14) and TCN1 (transcobalamin 1) were down-regulated genes in primary and metastatic tumors. On comparing probe sets from all 3 classes, we found that LMO3 (LIM-only protein 3) was the highest significantly up-regulated gene, while SLC6A14 was down-regulated the most amongst the genes. Further details and complete gene lists can be found in additional files.

**Fig. 4:**
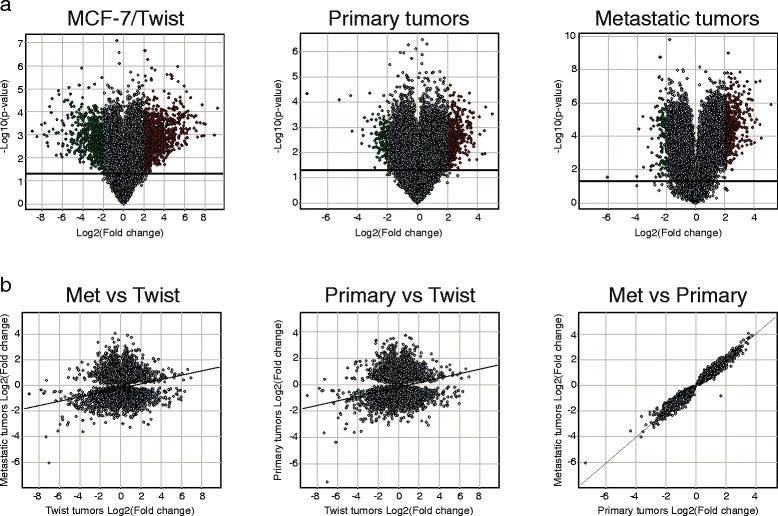
**a** Volcano plots displaying the expression of probe sets in MCF-7/Twist, primary tumors, and metastatic tumors compared to parental MCF-7 cells. Bold line at y = 1.3 corresponds to log10 value of significance (P = 0.05). Significantly up-regulated probe sets are colored red while significantly down-regulated probe sets are indicated in green **b** Scatter plots displaying the correlation between probe sets of various class comparisons - MCF-7/Twist, primary tumors, and metastatic tumors

To determine the variation in gene expression compared to controls, we displayed our microarray datasets with the help of volcano plots (Fig. 4a). Genes that were significantly different between the two comparison classes are displayed above the horizontal line (P < 0.05). As seen from the volcano plots, the maximum changes induced by Twist overexpression was in the MCF-7/Twist cell line, followed by the primary tumors, and lastly the metastatic tumors.

Finally, we compared the genetic changes caused by Twist expression in the breast cell line and in the orthotopic mouse xenograft and metastasis model. As seen in the scatter plots (Fig. 4b), the highest correlation is seen between the primary and metastatic tumors in mouse while much lower correlations were seen when comparing the MCF-7/Twist cell line data with the primary and metastatic tumor data.

### Genes dysregulated by Twist are observed in human breast cancer datasets

**Fig. 5:**
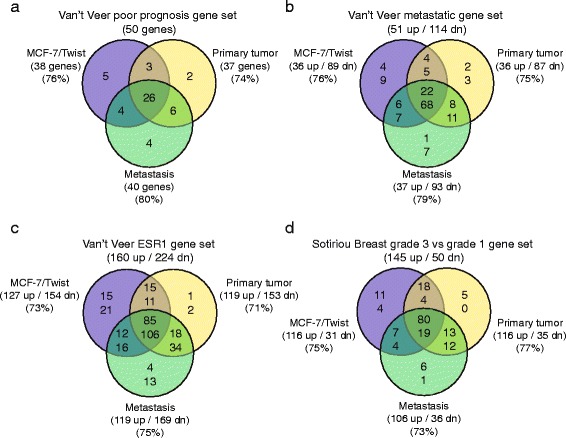
Venn diagrams displaying overlaps between MCF-7/Twist, MCF-7/Twist primary tumors, and MCF-7/Twist metastatic tumors datasets from the **a** van’t Veer Poor prognosis gene set, **b** Metastatic gene set, and **c**. Estrogen receptor gene set. **d** Our datasets compared to the Sotiriou “breast grade 3 vs grade 1 gene set”. Number of genes and direction of expression is indicated in parentheses along with percentage of overlap

To study how our Twist overexpressing cell line and orthotopic mouse primary and metastatic mouse model compared with human breast cancers, we analyzed data from the van’t Veer [23], Sotiriou [24], and Perou datasets [25]. From the van’t Veer “poor prognosis” dataset consisting of 50 genes, a total of 38, 37, and 40 genes were present in the MCF-7/Twist, primary, and metastatic datasets, respectively (Fig. 5a). Of these, 26 genes overlapped in all 3 classes. The van’t Veer “breast cancer up in metastasis gene set” was present in 36, 36, and 37 of 51 genes in MCF-7/Twist, primary, and metastatic datasets, respectively. In the other “breast cancer down in metastasis gene set” was present in 89, 87, and 93 of 114 genes in the respective datasets (Fig. 5b). Overall, the van’t Veer breast cancer metastasis dataset had 90 genes (22 up-regulated and 68 down-regulated) in common across all 3 of our datasets. From the van’t Veer “estrogen receptor positive breast cancer gene set” of 384 genes, we found an overlap of 281, 272, and 288 genes in our MCF-7/Twist, primary and metastatic datasets, respectively (Fig. 5c). This high degree of overlap corroborates our earlier work, which demonstrated that Twist expression correlates with and represses ER expression [4, 14]. Moreover, it is a validation of the role of Twist in breast cancer progression.

Additionally, we compared the Sotiriou dataset that has a gene signature that discriminates grade 3 from grade 1 breast tumors [24]. A total of 99 genes from our datasets overlapped with the Sotiriou dataset (195 genes) (Fig. 5d). The MCF-7/Twist, primary tumor, and metastatic tumor datasets had 147, 151, and 142 genes, respectively, overlapping with the Sotiriou dataset.

**Fig. 6:**
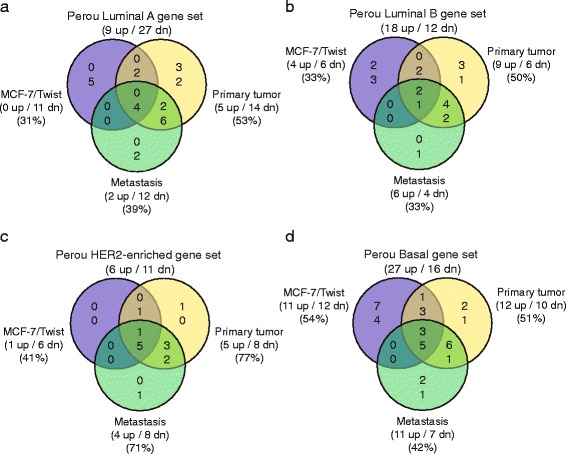
Venn diagrams comparing MCF-7/Twist, MCF-7/Twist primary tumors, and MCF-7/Twist lung metastatic datasets with the Perou breast cancer subtype classifier gene sets. **a** Luminal A gene set, **b** Luminal B gene set, **c** HER2-enriched gene set, **d** Basal gene set. Direction of gene expression, number of genes, and percentage of overlap are presented in parentheses

Lastly, we compared our datasets with the Perou gene sets that classify breast tumors into intrinsic sub-types [25]. As expected, we observed the least amount of overlapping genes when compared to the Luminal A and B datasets (Fig. 6a, b). Higher concordance (6 genes) between datasets was observed with the HER2 enriched dataset (Fig. 6c). As seen in Fig. 6d, the largest overlap was observed in the basal gene set with 8 genes being common between the 3 classes.

### Rho GTPase and IL-6 signaling pathways are upregulated by Twist

**Fig. 7:**
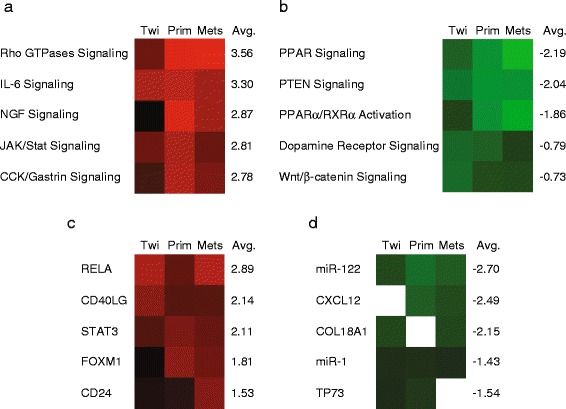
IPA analysis of datasets. **a**, **b** Heat maps of canonical pathways displaying average expression in MCF-7/Twist cells, primary tumors, and metastases. Red indicates up-regulation while green indicates down-regulation. **c**, **d** Heat maps of upstream gene regulators comparing expression in MCF-7/Twist cells, primary tumors, and metastases datasets

To determine the pathways that are dysregulated during Twist-induced breast cancer progression, we analyzed the datasets using the IPA (Qiagen) suite of software tools (Fig. 7 and Tables 1, 2).

**Table 1:** List of top 5 canonical pathways that are implicated in MCF-7/Twist cell line, MCF-7/Twist primary tumors, and MCF-7/Twist metastases

Canonical Pathways	*P*-value	Overlap (%)	Overlap (number of genes)
MCF-7/Twist cells			
Oncostatin M Signaling	3.29E-08	58.8%	20/34
Neuregulin Signaling	7.25E-08	41.2%	35/85
IL-8 Signaling	1.54E-07	32.2%	59/183
NRF2-mediated Oxidative Stress Response	1.69E-07	32.6%	57/175
GM-CSF Signaling	3.90E-07	44.3%	27/61
Primary tumors			
Role of NFAT in Cardiac Hypertrophy	2.38E-06	40.8%	71/174
Signaling by Rho Family GTPases	4.87E-06	38.1%	88/231
IGF-1 Signaling	5.96E-06	45.8%	44/96
α-Adrenergic Signaling	7.05E-06	47.1%	40/85
Dopamine-DARPP32 Feedback in cAMP Signaling	7.61E-06	40.8%	64/157
Metastases			
Role of Tissue Factor in Cancer	5.36E-07	34.6%	37/107
Molecular Mechanisms of Cancer	6.94E-06	24.0%	86/358
Role of NFAT in Cardiac Hypertrophy	8.39E-06	28.2%	49/174
CXCR4 Signaling	9.26E-06	29.1%	44/151
Pancreatic Adenocarcinoma Signaling	1.01E-05	32.1%	34/106

Pathways are ranked by *P*-value

**Table 2:** List of top 5 upstream regulator genes implicated in MCF-7/Twist cells, MCF-7/Twist primary tumors, and MCF-7/Twist metastases

Upstream Regulator Genes	P-value of overlap	Corrected z-score	Prediction
MCF-7/Twist cells			
Estrogen receptor	1.49E-18	−5.712	Inhibited
NUPR1	2.34E-14	1.843	
TP53	7.63E-14	−0.497	
TNF	7.66E-14	3.849	Activated
SMARCA4	9.56E-14	2.028	Activated
Primary tumors			
ERBB2	3.71E-06	0.231	
CD24	4.63E-06	1.199	
TP73	1.39E-05	−1.712	
TGFB1	2.48E-05	−0.534	
HSPA9	2.54E-05	−0.2	
Metastasis			
Lh	7.23E-05	−1.027	
CXCL12	1.54E-04	−2.071	Inhibited
NUP107	4.01E-04	1.052	
FOXA2	2.03E-03		
FSH	2.36E-03	−0.698	

Genes are ranked by *P*-value

Subsequently, when analyzed by IPA, the most significant pathways up-regulated in all three classes were signaling by Rho GTPases, IL-6, NGF, JAK/Stat, and CCK/Gastrin (Fig. 7a, b). On the other hand, PPAR, PTEN, PPARα/RXRα, dopamine receptor, and Wnt/β-catenin signaling were reduced. While comparing upstream regulators dysregulated in the three classes, we found RELA, CD40LG, STAT3, FOXM1, and CD24 the most upregulated. miR-122, CXCL12, COL18A1, miR-1, and TP73 were the top five upstream regulator molecules that were down-regulated in our dataset (Fig. 7c, d).

## Discussion

Metastases are the single largest cause of deaths from all cancers. The currently accepted model states that metastases arise when over a period of time a sub population of cells from the primary tumor acquire genetic alterations that confer upon them selective advantages that allow them to metastasize [26]. This widely belief has been challenged earlier [27, 28] and has recently come under increasing scrutiny due to microarray analysis of tumors and their associated metastasis [23, 29]. These studies demonstrate that the so-called “gene signature” of metastatic tumors is not very different from primary tumors. This implies that the genetic alterations that lead to metastasis are acquired much earlier than is presently anticipated - perhaps during formation of the primary tumor itself. The lack of progress in treatment of metastases is partly caused by the scarcity of clinically relevant animal models of breast cancer and metastases [30, 31].

We have earlier demonstrated that the Twist gene is involved in the progression of breast cancers towards a highly aggressive phenotype [4, 12, 14]. In this work, we over expressed Twist in non-metastatic MCF-7 and orthotopically implanted these cells in mouse mammary fat pads. After resecting the primary tumors, we monitored the animals for the development of metastatic lesions, primarily in lungs. Unlike other studies that directly assayed gene changes from primary and metastatic sites, we grew the cells out in plastic for 1–2 weeks before microarray analysis. This allows for us to lessen the impact of mouse cells that could have infiltrated the orthotopic tumors. Moreover, this also eliminates any fibroblast cells that could be carried over from the metastatic sites in lungs. To lessen batch variation, we used the same gene arrays (Affymetrix Human Genome HGU133 Plus 2.0) and the same core facility for all the work in this study. We also analyzed human breast cancers in our study. However, due to the limited sample size, our study was not powered enough to draw conclusions from this part of the study. Instead, we compared our Twist model with currently accepted human gene signature databases ([23–25, 32].

As expected, our model showed a high species-dependent correlation. The primary and metastatic tumors clustered strongly both in PCA analysis as well as by unsupervised hierarchical clustering. Strong clustering was also seen in the breast cancer samples. The PCA distribution of MCF-7 and MCF-7/Twist showed a higher than expected degree of separation. This could be due to the very strong effect of Twist on the MCF-7 cell line, which becomes ER-negative after Twist overexpression [14]. Over time, as tumors establish in mice, the effects of Twist may be ameliorated due to selection pressure from the various other dysregulated pathways of tumor growth.

In our model, the largest number of genes were dysregulated in the metastatic tumor class followed by the primary tumors, and least in the MCF-7/Twist class. This was expected since one could deduce that metastatic breast tumors are genetically the furthest away from the parental MCF-7 cell line, which formed the baseline of all the comparisons. We also observed that a high number of genes were commonly dysregulated in the primary and metastatic tumors. However, we also observed that MCF-7/Twist cells had a higher number of genes in common with the metastatic tumor class as compared to the primary tumors. This would indicate that the MCF-7/Twist cell line in vitro was a better representative of the metastatic process in mice than the primary tumors. When comparing the classes for genes that were expressed in opposite directions, as expected, we only found a significant number of genes when MCF-7/Twist was compared with the primary and metastatic tumor classes (either together or singly). This was further corroborated by quantile plots, which clearly indicated the close relationship between the primary breast tumors and the metastasis.

When comparing our datasets with the van’t Veer poor prognosis gene set, we observed that a large proportion of genes from all 3 classes overlapped with that dataset leading us to hypothesize that our model does recapitulate, in some measure, the progression of breast cancer in humans. Similarly, the Sotiriou gene set also exhibited a high degree of concordance with our dataset implying that the Twist model is a suitable model system for high-grade breast tumors. When comparing the Perou dataset of intrinsic sub-types of breast cancer, we observed that our datasets overlapped ﻿the most with the basal and HER-2 enriched datasets. A very low degree of overlap was seen with the Luminal A and Luminal B sub-types.

To model which biological functions and pathways were dysregulated by Twist overexpression, we employed IPA analysis. Overall, we saw an increase in transcription and translational activity, and involving genes related to cellular biogenesis and apoptosis. Also, enriched were genes in the protease family and extracellular matrix. This was expected since Twist is a master transcription factor for many pathways and is also involved in apoptosis and in modulating the extracellular matrix to cause invasion and metastasis.

## Conclusions

In summary, we generated Twist-driven models of breast cancer progression, both in vitro and in vivo, that mimic the ontology of breast cancer formation and metastatic development in humans. These datasets will help in identifying Twist-associated dysregulated genes and pathways that can be targeted to prevent or decrease breast cancer metastases. Importantly, our work provides evidence that gene-specific functions can be ascertained by using preclinical models that can be associated with the ontogeny of human breast cancer formation and progression.

## Additional files

Additional file 1: Ranked list of significant probe sets up and down-regulated in MCF-7/Twist cells, primary tumors, and metastases. Probe sets are ranked by average fold change across all 3 groups. (XLSX 148 kb)
Additional file 2: Histogram displaying results of validation of genes up-regulated (LMO3 and BICC1) and down-regulated (DSCAM-AS1 and SLC6A14) in the MCF-7/Twist cell line, primary tumors, and metastases. (EPS 1432 kb)
Additional file 3: Ranked list of probe sets expressed multi-directionally in all three groups. (XLSX 22 kb)
Additional file 4: Ranked list of significant probe sets most differentially expressed in MCF-7/Twist cells. Probe sets are ranked by fold change. (XLSX 461 kb)
Additional file 5: Histogram displaying results of qRT-PCR validation of genes up-regulated (EDIL3 and LDHB), and down-regulated (DSCAM-AS1 and NPY1R) in the MCF-7/Twist cell line. (EPS 1258 kb)
Additional file 6: Ranked list of significant probe sets most differentially expressed in MCF-7/Twist primary tumors. Probe sets are ranked by fold change. (XLSX 601 kb)
Additional file 7: Histogram of qRT-PCR validation of genes up-regulated (SETD5 and TFAP2A) and down-regulated (SLC6A14 and LINC01419) in MCF-7/Twist primary tumors. (EPS 1253 kb)
Additional file 8: Ranked list of significant probe sets differentially expressed in MCF-7/Twist lung metastases. Probe sets are ranked by fold change. (XLSX 734 kb)
Additional file 9: qRT-PCR validation results of genes up-regulated (SETD5 and TFAP2A) and down-regulated (SLC6A14 and HPGD) in MCF-7/Twist lung metastases. (EPS 1253 kb)
Additional file 10: Ranked list of significant probe sets up and down-regulated in MCF-7/Twist primary tumors and MCF-7/Twist lung metastases. Probe sets are ranked by fold change. (XLSX 740 kb)
Additional file 11: qRT-PCR analysis of genes up-regulated (SETD5 and TFAP2A), and down-regulated (NR2F1 and GJA1) in MCF-7/Twist primary xenograft tumors and in lung metastases. (EPS 1264 kb)
Additional file 12: Ranked list of significant probe sets up and down-regulated in both MCF-7/Twist cells and MCF-7/Twist lung metastases. Probe sets are ranked by fold change. (XLSX 38 kb)
Additional file 13: Histogram showing qRT-PCR validation of genes up-regulated (EIF1AY and PLAU) and down-regulated (WDR72 and AGR2) in MCF-7/Twist cell line and in lung metastases. (EPS 1266 kb)
Additional file 14: Ranked list of significant probe sets up and down-regulated in both MCF-7/Twist cells and MCF-7/Twist primary tumors. Probe sets are ranked by fold change. (XLSX 32 kb)
Additional file 15: Histogram displaying qRT-PCR validation results of up-regulated genes (POPDC3 and CXADR) and down-regulated genes (NPY1R and IGFBP5) in MCF-7/Twist cells and primary tumors. (EPS 1268 kb)

## Abbreviations

RMA: Robust multi-array Average, qRT-PCR (quantitative reverse transcription polymerase chain reaction)
